# Type 1 Diabetes and Other Autoimmune Disorders in Children

**DOI:** 10.1155/2024/5082064

**Published:** 2024-02-26

**Authors:** Eleonora Agata Grasso, Francesco Chiarelli

**Affiliations:** Department of Paediatrics, University of Chieti, Via dei Vestini 31, Chieti, Italy

## Abstract

The incidence of autoimmune disorders (AIDs) has been dramatically increasing in both children and adults over the past few years, and type 1 diabetes (T1D) is one of the diseases that has seen the highest growth. It is well-known that the dysimmune process may spread to other systems, leading to the onset of one or more AIDs in the same individual; however, the relationship between AIDs is not often recognized. The most frequently diagnosed AIDs in children and adolescents with T1D are thyroid diseases and celiac disease, but it is also important to consider the onset of the other conditions, such as juvenile idiopathic arthritis, multiple sclerosis, atrophic gastritis, inflammatory bowel diseases, and skin disorders such as vitiligo and psoriasis. This review aims to explore the overlap of T1D and other AIDs, focusing on the less common and lesser-known diseases. A better knowledge of these comorbidities may facilitate the identification of patients at risk to treat them in the preclinical period, before the onset of complications.

## 1. Introduction

The incidence of autoimmune disorders (AIDs) has been dramatically increasing both in children and adults over the last 30 years. Indeed, the prevalence of type 1 diabetes (T1D) mellitus in children is estimated to grow by 2%–5% every year worldwide, albeit with some differences between geographic locations [1–4].

Since the autoimmune reaction may spread to the other systems, resulting in comorbidities, the onset of additional AIDs in the same individual over the course of T1D has been an object of interest, suggesting a strongly shared genetic susceptibility and shared pathological mechanisms with other AIDs [5]. To support the spread of autoimmunity over disease-specific boundaries is the formation of alternative autoantibodies associated with a different disease classification in the set of a specific AID [6]. This process is called “poly-autoimmunity,” and was suggested to have a role in patients' subsequent clinical presentation and progression [7].

Pediatric endocrinologists are already used to keeping an eye open for issues such as thyroid dysfunction and celiac disease (CD) [8], but the rapid rise in prevalence of autoimmunity—although with different distribution in diverse geographic locations and among different ethnicities—brings into question the overlap and coexistence with other less well-known autoimmune diseases (Table 1) [39]. Although, the pathology of each of these disorders is diverse, genetic, and environmental factors are often shared in the different AIDs. Hence, the genetic predisposition and the influence of the environment in the regulation of the immune system may help to explain the reasons for their increased prevalence [7].

The aim of this review is to explore the relationship and coexistence between T1D with other AIDs, ranging from the most frequent and known disorders to the ones that are less common but still equally important.

## 2. Materials and Methods

We analyzed the relevant Literature published between 2010 and 2023, some work before 2010 has also been included for the significant scientific impact on the topic. Electronic databases (MEDLINE via PubMed, EMBASE) and original scientific papers were systematically searched for “type 1 diabetes AND autoimmune disorders”. A subsequent individual search for each comorbidity has been performed (e.g., “type 1 diabetes AND juvenile arthritis”, “type 1 diabetes AND celiac disease”). Once the articles have been identified a thorough review of all results was conducted, selecting papers that were relevant for pediatric age (below 21 years of age). Only articles written in English have been included in the search. Results and conclusions were compiled and summarized.

### 2.1. Risk Factors and Mechanisms of Pediatric Autoimmunity

The rise of AIDs has given interest to their epidemiology and the risk factors related to them [40].

The global prevalence of AIDs in children is about 5%, with the most frequent ones being autoimmune thyroid diseases and T1D [36, 40], the latter of which has an estimated prevalence of 1 case per 300 children in the United States [2]. However, a study of 179 children with diabetes reported that 15.6% of the cohort was positive for additional autoantibodies (anti-thyroid peroxidase, anti-thyroglobulin, or anti-transglutaminase antibodies) and 2.8% already had another AID at the time of the diagnosis of T1D [5]. Moreover, the incidence of both antibody positivity and the onset of additional AIDs grew progressively during follow-up.

The development of an AID may follow a defective suppression of T-cells' reaction against self-antigens (“suppressor hypothesis”), and/or the formation of autoreactive autoantigen-specific lymphocytes (“effector hypothesis”) which may cause the secretion of autoantibodies and the manifestation of the disease in predisposed individuals [41].

**Figure 1:**
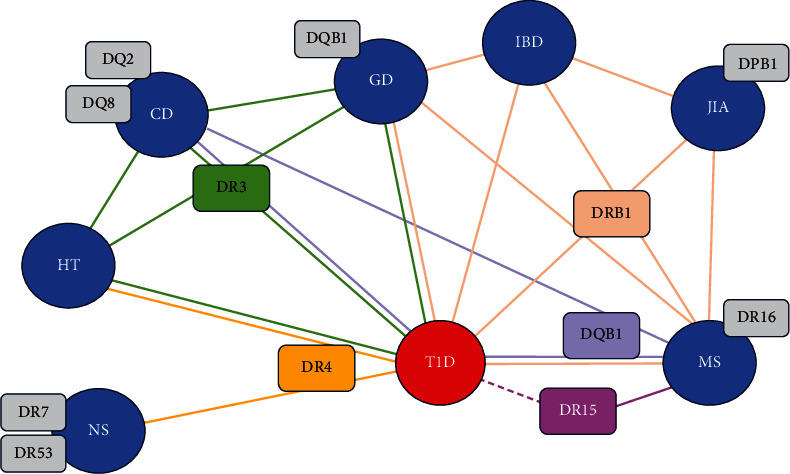
Summary of the major associations within the HLA class II with autoimmune diseases. Abbreviations. CD, celiac disease; GD, graves disease; HT, hashimoto thyroiditis; IBD, inflammatory bowel diseases; JIA, juvenile idiopathic arthritis; MS, multiple sclerosis; T1D, Type 1 diabetes; NS, nephrotic syndrome (Minimal change disease). Unbroken lines represent the increased susceptibility to the disease having the HLA; broken lines represent the protective effect of HLA on the development of the disease.

Genetics is under the spotlight to explain the invisible link between some autoimmune phenotypes, as supported by the co-occurrence of one or more conditions in the same patient and within the same populations and families [42]. Pragmatic examples are given by the onset of autoimmune X-linked poly-endocrinopathy in patients with a mutation in FOXP3—a transcription factor known to modulate the development of regulatory T-cells—or in cases of autoimmune polyendocrine syndrome type 1 (APS1), where the mutation in the gene AIRE interfere with FOXP3 expression [42]. Other genes associated with autoimmunity are the one of the human leukocyte antigen (HLA) system, the CTLA4, and PTPN22, since they all positively or negatively regulate the activation of T-cells [43]. HLA loci have been linked with different conditions, thus identifying overlaps may help to understand the onset of comorbidities (Figure 1). For example, DBQ1, DR3-DQ2, and DR4-DQ8 are the primary determinants for T1D [1], but DR3-DQ2 also predisposes to CD, and the risk of having transglutaminase antibodies in homozygote T1D patients is estimated to be equal to 33% [44]. On the other hand, many loci have been defined as protective for some of these conditions, such as DR15-DQB1 ^*∗*^0602 and DR1301 for T1D. However, the more frequent onset of diseases in patients “genetically protected” favors the notion that HLA-linked genetic susceptibility may decrease and/or the environmental pressure may increase [45].

Among the non-HLA loci, the CTLA4 gene, located on chromosome 2q3, and its associated protein are important negative regulators of T-cell activation and have been associated with the onset of T1D and autoimmune thyroiditis [46]. Finally, PTPN22 (chromosome 1p13) encodes for LYP, a protein expressed in lymphocytes with a negative regulatory role. Polymorphism of this gene has been linked to T1D, autoimmune thyroiditis, and juvenile idiopathic arthritis (JIA) [43].

Genome-wide association studies also demonstrated the presence of numerous single nucleotide polymorphisms (SNPs) linked with the onset of some AIDs, although they may not have a functional role, they are in linkage disequilibrium with the variants that are themselves causal [42].

Aside from genetics, several other determinants are being considered to explain the onset of AIDs (Table 2), as also supported by the wide variability of AIDs in monozygotic twins, estimated to range from 12% to 67% [47].

In genetically predisposed children, environmental triggers may stimulate the hyperexpression of MHC class I and II, leading to CD8+, macrophages, and B-cells activation first, and CD4+ helper later. Epigenetics mechanisms are associated with loss of immune tolerance through hyper or hypomethylation of DNA (e.g., hypomethylation of peptidyl arginine deaminase 2 and Src homology region 2 domain-containing phosphatase-1 in MS, or methylation of the proximal insulin gene promoter in T1D [48]).

Another point of interest is the geographic prevalence of AIDs; since some countries report a higher prevalence for many of these conditions [36, 49], which cannot be explained by the genetics alone. Indeed, migrant studies proved how the risk of developing AIDs may vary moving from a region with a lower prevalence to one with a higher one [50, 51]. As such, rates of T1D are higher in some European countries (e.g., Finland and Italy) [12] compared to Asia where the incidence rates are usually very low [52]. Similarly, MS is more prevalent in countries located at higher latitudes, such as Canada, northern countries of Europe and the USA, and Southern Australia [53]. To explain such regional differences, many authors identified UV light exposure and, consequently, vitamin D activation as suitable answers to this question. Although data about the pathogenic role of vitamin D deficiency are controversial, many studies support its supplementation in the prevention of AIDs and/or reduction in disease flairs with limited health risks [54].

A female preponderance is seen in many AIDs and may be related to X-linked genetic factors or reproductive hormonal factors—as supported by the more equal distribution between males and females before puberty in some AIDs [7, 55, 56]. Among these, T1D does not display a strong female bias [57], although the incidence of AIDs may display variations among genders in different geographic locations. Under 15 years of age, it is described a minor male prevalence in the European population versus a female predominance in populations of African and Asian origin [57, 58]. Interestingly, all populations with a higher incidence of T1D display a male preponderance, whereas all those with a low incidence (lower than 4.5/100,000) have a female excess [57]. A similar fashion has been described also for Crohn's disease, for which is also reported a male predominance in certain areas and a reverse correlation in Asian countries [59, 60]. However, the reason for such variations is still unknown.

Infections are one of the most known triggers for autoimmunity, with several germs implied on the onset of various AIDs. Antigens of viruses and bacteria may resemble selected antigenic determinants of the host, leading to T-cell activation. This process is called “molecular mimicry” and has been described in several conditions such as acute rheumatic fever following a Streptococcus Pyogenes' infection, or Epstein-Barr virus which was linked to MS, SLE, rheumatoid arthritis, Sjogren syndrome, and primary biliary cholangitis [48, 49]. Notably, an increased prevalence of autoimmune conditions has been reported also following the COVID-19 pandemic [61]: SARS-CoV-2 was found to determine hyperstimulation of the immune system, with the induction of the so-called “cytokines storm”, characterized by an increased release of inflammatory cytokines (e.g., IL-6), and of an excessive neutrophil extracellular traps activation, a process known to be involved in the abnormal inflammatory response described in SLE, rheumatoid arthritis, and MS [61].

The higher frequency of AIDs in industrialized countries has been related to reduced exposure to infections, consequent to the improvement of hygienic conditions. Indeed, the “hygiene hypothesis” suggests that the decrease of the infection burden has led to a Th1–Th2 deviation and to a lack of development of strong immune responses toward germs, which would train the immune system to avoid reacting toward autoantigens and allergens [62].

Another interesting aspect involves the correlation between BMI, diet, and microbiota.

Obesity is known to be linked to a low-grade systemic inflammatory state, which may impact the risk of developing AIDs either during childhood or at an older age [63, 64]. In addition to the higher risk of developing AIDs (such as MS, IBDs, T1D, and rheumatic diseases) in obese patients, disease activity and a lack of response to the therapies were found to be proportional to the BMI of the patients, supporting the importance of weight loss or weight control interventions as adjunctive therapy for patients with obesity and AIDs [65–68].

Environmental changes and modifications of infant and maternal diets may be some of the possible factors that have an impact on the development of AIDs. Maternal antibodies can attenuate the risk effect of specific viruses in children who are breastfed, giving a suggestive explanation for the lower incidence of AIDs as T1D in low-income countries where prolonged breastfeeding is more common [69]. However, studies about maternal diets and breastfeeding are conflicting [70]. Among the intrauterine factors, the placental transmission of viruses has been correlated with T1D (e.g., rubella) [3]. Furthermore, preterm births have been associated with both T1D, JIA, and autoimmune thyroid diseases. This can be explained by high-maternal age, reduced exposition to the maternal environment (e.g., antibodies, placenta-derived elements), higher use of antibiotics in preterm babies, and alteration of the neonates' microbiota [71].

Gut microbiota plays an important role in regulating the host immune response through the activation of antigen-presenting cells and the production of cytokines, affecting T-cell differentiation and function. Gut dysbiosis has been described in many diseases, despite it is not certain if it may be causative of the disease or a consequence of the inflammatory state, especially in CD and IBDs [6, 72–79]. Interestingly, several AIDs present with a similar pattern of alteration (e.g., psoriasis and IBDs [80], T1D and, CD [81]); one may speculate that these overlaps may have clinical consequences, despite further studies being needed to assess this. Moreover, the role of probiotics as a tool to modulate the gut microbiome to an anti-inflammatory state needs further investigation [73].

### 2.2. Common Comorbidities of T1D

#### 2.2.1. Thyroid Diseases

Thyroid disorders are among the most frequent endocrinological disorders recognized in the pediatric age groups, with an overall incidence of autoimmune hypothyroidism of 1%–3% in the United States, which is overall significantly more frequent than hyperthyroidism, which constitutes only 15% of children's thyroid disorders [21]. However, in a cohort of 808 patients in Taiwan, 4.1% of patients developed Graves' disease compared to 1.4% of patients who had Hashimoto disease [19].

The association between T1D and thyroid disease has been frequently reported, estimated to be 15%–30% [24–27, 31]. Thus, the American Academy of Diabetes recommends screening for autoimmune thyroid disease soon after the diagnosis, and periodically thereafter [82]. The overlap between these two conditions is also found in the polyglandular syndrome type 2, typically represented by Addison's disease (AD), autoimmune thyroid disease, and/or T1D.

Therefore, National and International Guidelines for diabetes management recommend screening for anti-thyroid peroxidase and anti-transglutaminase antibodies, TSH, and free T4 at the onset of T1D—once the child is clinically stable—and to monitor thyroid function during the lifetime of these patients [39, 83, 84]. At the onset of diabetes, 25% of children are thyroid-antibodies positive. Among these, thyroid peroxidase antibodies are more predictive than anti-thyroglobulin antibodies [39].

Timing-wise, it is suggested to check the TSH every 1–2 years, or sooner if the patient has positive thyroid antibodies or presents with signs or symptoms that may be related to thyroid dysfunction, such as thyromegaly or abnormal growth rate [39]. Notably, thyroid dysfunction may also impact negatively metabolic parameters (HbA1c, LDL, HDL, and triglycerides) and increase the number of episodes of hypoglycemia, so an unexplained glycemic variability may be a subtle sign of thyroid disease [39].

#### 2.2.2. Celiac Disease

CD is diagnosed in 4.6%–16.4% of T1D patients, and—similarly to T1D—its incidence has increased progressively in the last 30 years, though the incidence rate is starting to level off [12–16]. As previously mentioned, mutual genetic risk factors explain the higher chance of developing both conditions [85–87]. As for other AIDs, both CD and T1D have been linked to preceding infections and other environmental factors such as socioeconomic status, gut microbiota disbalance, and vitamin D deficiency [15]. Whereas in T1D the trigger for the onset of the immune cascade is unknown, dietary gluten has been identified as the causative antigen for CD. Some studies questioned the beneficial role of gluten-free diets in preventing other AIDs such as T1D, MS, and autoimmune thyroid diseases, although there is currently little evidence to support this diet for non-celiac patients [88].

The diagnosis of both CD and T1D relies on antibodies, although transglutaminase antibodies have 100% positive predictive value for CD, compared to only 10%–25% for a single antibody positivity in patients with T1D. Still, it increases to 75% in cases of positivity for multiple antibodies [89]. Both diseases may have a long subclinical phase that reflects the slowly progressive damage of the bowel and the pancreas, respectively. Since, T1D may present with more subtle symptoms than the characteristic weight loss, thirst, hunger, and polyuria, and CD may express a wide spectrum of extraintestinal symptoms (e.g., poor growth, aphthous ulcers, dermatitis herpetiformis, joint disorders), diagnosing one condition in the context of the other can be tricky and lead to a significant delay in the diagnosis, since many symptoms are sometimes overlooked or assumed to be linked to the other known issue. This was proved by a study conducted on 118 patients with both T1D and CD, which reported a 5-year delay in CD diagnosis in 33% of patients; the authors emphasize the importance of dosing the antibodies when in doubt [90].

Therefore, the screening for CD relies on measuring IgA tissue transglutaminase and assessing normal IgA levels (or IgG transglutaminase if IgA deficient) after the diagnosis of T1D and then again within the first 2 years of disease [82]. The antibodies should be dosed again after 5 years unless the patient develops signs suggestive of CD (diarrhea, malabsorption, abdominal pain, osteoporosis, iron deficiency anemia, or vitamin deficiency), which would require the anticipation of the screening [39].

### 2.3. T1D and Other AIDs

#### 2.3.1. Rheumatological Disorders

Some studies suggest a higher risk of developing T1D in patients with JIA, with an estimated prevalence of 0.5% [29]. On the other hand, JIA prevalence in children was 0.19%, compared to 0.05% in the general population [29, 30]. Genetic variations of genes that cause T-cell dysfunction (e.g., HLA, CTLA4, and PTPN22) may be involved in the development of these conditions [91]. On the other hand, diabetes may be facilitated by the adverse effects of frequent corticosteroid use on JIA flairs [92].

A Finnish study explored the incidence of T1D and JIA together over three decades; 82 patients were identified and the co-occurrence of the two diseases increased over the observation period [93]. Among the subtypes of arthritis, 15% were rheumatoid factor seropositive, and only a small portion of the cohort had systemic arthritis. Only 7% of patients (*n* = 6) had uveitis. Interestingly, 1/5 of the patients had a third autoimmune disease (e.g., CD or hypothyroidism). Similarly, a study on a large cohort of children aged between 6 and 18 years old was performed to assess the prevalence of T1D [94]. The cohort was made up of three groups: JIA, asthmatic, and healthy children. The incidence rate of T1D was the highest among the patients with JIA, with 44 cases for 100,000 person-years, compared to 29 cases in the asthma subgroup and 22 in the healthy cohort. Interestingly, this rate was lower among JIA patients with a history of use of disease-modifying drugs. Hence, the immune-modulatory effect may have a role in preventing the immune cascade responsible for the onset of T1D, suggesting that an earlier start of these effective drugs may have a protective effect on beta cell preservation. Moreover, the use of disease-modifying antirheumatic drugs has been associated with a significant improvement in HbA1c and a reduced need for insulin [92], hence these therapies in JIA may impact the risk of developing T1D and/or improve T1D disease course [95–99].

Although to a lesser extent compared to JIA, systemic lupus erythematosus has been described as a comorbidity of T1D. This overlap is supported by the shared haplotypes HLA-DR3 and DR4, and the shared disease susceptibility alleles STAT4 and TNFAI3P [95–99]. Polyautoimmunity was described in about 9% of patients from a large cohort of patients with childhood-onset SLE (*n* = 1,285). The most frequent autoimmune comorbidities were thyroid disease (in 10 patients, 2.5%), whereas one patient had CD and another one T1D (0.26%) [100]. Another study conducted in Saudi inquired about the incidence of endocrinopathies in 42 patients with JIA or SLE; respectively, four patients with JIA and one with SLE were also diagnosed with T1D [101]. Other organ-specific antibodies are frequently found in patients with SLE, although the frequency of T1D-associated antibodies has not been largely established. In a cohort of 41 patients with SLE, 5% of patients were found to have T1D antibodies and two patients fulfilled the criteria to be diagnosed with diabetes [102]. The overlap between SLE and T1D is also supported by the shared haplotypes HLA-DR3 and DR4, and the shared disease susceptibility alleles STAT4 and TNFAI3P [95–99]. The coexistence of SLE with T1D has been linked to renal failure [96], an unfavorable outcome shared by both conditions. Therefore, in a patient with T1D and features of connective tissue disease the etiology of the renal disease is based on the results of a biopsy [96].

On that account, more studies are needed to assess the prevalence of rheumatic comorbidities in T1D and the role of antibodies' screening for organ-specific diseases in patients with JIA or SLE.

#### 2.3.2. Neurological Disorders

The overlap between AIDs and neurological disorders has been thoroughly studied in recent years, suggesting a role in the inflammatory process of diseases for epileptogenesis [103, 104]. T1D usually precedes epilepsy, although a cohort study reported a higher incidence of diabetes in patients with refractory epilepsy [105, 106]. Despite many large cohort studies of patients with AIDs reporting an increased prevalence of epilepsy, a clear association between these two diseases has not been established [103, 107]. Among the markers studied to explain the link between diabetes and epilepsy, GAD65 antibodies were found to decrease the conversion of glutamic acid to GABA, compromising the balance between excitatory and inhibitory neurotransmitters and leading to hyper-excitability. In a cohort of nine patients who tested positive for antineuronal antibodies, eight carried GAD65 antibodies [108]. These antibodies have been found in a subgroup of patients affected by autoimmune encephalitis, defined by the presence of neurological dysfunction, evidence of inflammation in the central nervous system, and exclusion of alternative diagnosis [109]. The clinical manifestations may vary and include limbic encephalitis, epilepsy, cerebral ataxia, and stiff-man syndrome [110]. Interestingly, the onset of neurological symptoms is associated with the titers of GAD65 that are 100–1,000 times higher than the ones usually seen in patients with T1D [109]. To confirm the pathogenical role of GAD65 in the onset of neurological symptoms it may also be useful the search for these antibodies in the cerebrospinal fluid [111].

Aside from epilepsy, many studies focused their attention on autoimmune demyelinating disorders, especially on the relationship between T1D and MS [49]. Both conditions are T-cell mediated with strong involvement of the B-cell compartment, as supported by the positivity for insulin antibodies in children with T1D and the presence of oligoclonal bands in patients with MS. Certain haplotypes within the HLA system as well as SNPs may genetically contribute to the susceptibility for both diseases [112, 113], but other environmental factors (e.g., viral infections, seasonality, vitamin D exposure) may come into play [49, 114]. Despite the rarity of pediatric-onset MS compared to T1D, several reports reported the co-occurrence of these conditions, with a threefold to fivefold higher prevalence of T1D in patients with MS [115]. A study conducted on 248 diabetes centers in Germany and 13 centers in Austria initially identified 19 patients with MS in a cohort of 56,653 children and adolescents with T1D, demonstrating around a 3–5 times higher rate of patients with MS in the T1D cohort compared to the general population [116].

Similarly to JIA, there is evidence that targeting B cells with anti-CD20 monoclonal antibody, widely used in MS, can delay the fall of C-peptide by months [117].

Hence, T1D and MS share some similarities that explain the overlap between the two conditions. Children and adolescents affected by MS are routinely monitored with blood tests, despite an urgent screening for T1D is necessary in case of the onset of symptoms suggestive of T1D.

A deeper understanding of their commonalities may accelerate progress in achieving an early diagnosis and in tailoring the management.

#### 2.3.3. Atrophic Gastritis and Inflammatory Bowel Diseases

Autoimmune atrophic gastritis is the result of the formation of antibodies against parietal cells and intrinsic factors leading to mucosal destruction. Its association with other AIDs has been documented, such as thyroid disease, T1D, and vitiligo. Indeed, in a cohort of 34 patients, 32 had concomitant AIDs, 25 (74%) had thyroid disease, 19 (65%) had T1D and 8 (9%) had vitiligo [118]. Although, the co-occurrence of autoimmune atrophic gastritis and T1D is common in adults, pediatric cases are limited [119–121]. Interestingly, among the cases reported, a 15-year-old girl had also Graves's disease since the age of 13 and T1D from age 6 [121]. Another girl of the same age had hypothyroidism since the age of 8 years. In all the cases described so far, atrophic gastritis was diagnosed after ruling out the causes of anemia, and the authors noted that doctors should keep this condition in mind when approaching a child with diabetes and anemia of unknown origin.

Similarly to the other AIDs, in the past two decades, inflammatory bowel diseases (IBDs) have been more prevalent in children, with an incidence of 10 patients for every 100,000 children in the United States and Canada [122, 123]. Data about the associations between T1D and IBDs are limited and often conflicting [124, 125]. In a cohort of 65,147 children with diabetes, the prevalence of IBDs was higher than in the general population of the same age group and estimated to be about 0.1% [126]. The study also brings to light the fact that patients suffering from both diseases have a higher risk of severe hypoglycemia, most likely due to the consequences of malabsorption consequent to bowel inflammation. Patients with IBD and diabetes are also exposed to higher morbidity and mortality compared to IBD alone [127, 128]. Some studies have suggested that many mechanisms may explain these findings, such as the alteration of the gut microbiome, chronic systemic inflammation, and overlapping immune activity [126].

Therefore, even though the prevalence of both disorders does not seem to be relevant compared to the other AIDs, it is important to keep in mind autoimmune atrophic gastritis and IBD, respectively, in children with T1D and anemia of unknown origin or gastrointestinal symptoms.

#### 2.3.4. Nephrotic Syndrome

Idiopathic nephrotic syndrome is the most common type of renal disease in children, and it is characterized by a daily loss of protein in the urine, hypoalbuminemia, and the presence of edema [129].

On the other hand, signs and symptoms of kidney damage throughout diabetes are usually the consequences of diabetic nephropathy, a well-known chronic complication of T1D, which is usually associated with hypertension and retinopathy. However, several cases of idiopathic nephrotic syndrome in the set of T1D have been reported [130], and the mutual immune roots support a noncoincidental association. Despite the exact mechanism being unknown, many case reports suggest a mutual genetic component given an overlap for HLA in many patients, such as DR4, DR7, and DR53 [130–134].

A short period of T1D and the absence of other organ damage (e.g., retinopathy) supports the differential diagnosis of diabetic nephropathy. Kidney biopsy may help to distinguish the two conditions and it usually detects minimal focal glomerulonephritis and none of the abnormalities suggestive of diabetic nephropathy [135].

Children with T1D and idiopathic nephrotic syndrome usually respond to the administration of corticosteroids, with loss of edema, as well as resolution of proteinuria and serum abnormalities [135]. A favorable effect of rituximab has been described, as well as a good glycemic profile despite chronic steroid use, probably due to the steroid-sparing effect of this drug [134]. Moreover, immunosuppressive drugs such as rituximab showed a beneficial effect in T1D patients [117] and the anti-CD3 drug teplizumab is being evaluated by the Food and Drug Administration as a first disease-modifying therapy for T1D [136, 137].

Although, diabetic nephropathy is the leading cause of renal disease in T1D, idiopathic nephrotic syndrome must be considered in the differentials when symptoms onset after a short time from the diagnosis and a normal renal function right before the onset of symptoms.

#### 2.3.5. Skin Disorders

Diabetes systemic effects may translate into a wide spectrum of dermatological disorders. Although, most of the most frequent signs and symptoms are seen in type 2 diabetes and are related to insulin resistance, some typical lesions may occur in T1D as well, such as necrobiosis lipoidica diabeticorum or diabetic dermopathy [138, 139]. Even though there is wider evidence for a link between psoriasis and type 2 diabetes, this condition may also be related to T1D [32, 140], especially in obese patients [141]. The prevalence of psoriasis was evaluated in a cohort of 166 children, finding a fourfold increase compared to the general population [33]. The authors also reported higher levels of HbA1c at the onset of psoriasis, suggesting a role for hyperglycemia in the development of this condition. The link between hyperglycemia exposure and psoriasis may be explained by the formation of the advanced glycation end products (AGEs), which could play a role in the dysregulation of keratinocytes' apoptosis [32]. This was supported by the detection of high levels of AGEs in the skin and blood of patients with severe psoriasis [142]. Moreover, higher levels of adipokines in obese patients interfere with the inflammatory cytokines (e.g., IL-23, IL-17, and IL-18) involved in the development of either T1D or psoriasis [141]. Interestingly, treatment with alefacept, a biologic immunosuppressive drug currently prescribed for psoriasis that targets CD4 and CD8, resulted in quantitative and qualitative changes in effector T cells and, consequently, in prolonged preservation of beta-cells in patients with new-onset T1D [143, 144].

Vitiligo is a common dermatological disorder characterized by an acquired appearance of white macules on the skin, hair, and mucosa. Even though the etiology of this condition is not fully known yet, autoimmune pathogenesis has been hypothesized, suggesting that an autoimmune dysregulation may lead to melanocyte damage [145]. The overlap of vitiligo with AIDs supports this possibility, as described in cases of Graves' disease, autoimmune gastritis, adrenocortical insufficiency, and idiopathic hypoparathyroidism [37, 146–149]. Vitiligo has a 2%–10% prevalence in patients with T1D [36]. In a cohort of 300 young patients with T1D, vitiligo was found in five patients, with a prevalence of 1.6%, compared to the 0.7% of the general population [37]. The association between vitiligo and T1D has been addressed by studies that focused on the role of CD-4 positive T-cells in both diseases [150, 151], although a specific target common to both conditions has not been found yet.

Another skin disorder which has been described in patients with T1D is alopecia areata. Despite the pathophysiology of this condition is not fully understood, alopecia areata is the result of an autoimmune attack toward the hair follicles, which causes hair loss in one or more focal regions, the complete scalp, or—in worse cases—the entire body [152]. Patients affected often present with other AIDs, such as vitiligo, thyroid diseases, autoimmune gastritis, AD, or T1D [153–157]. Interestingly, it was reported that hair loss may improve after reaching good glycemic control, suggesting that a metabolic derangement may also be implicated in the pathology of the alopecia [158].

Finally, chronic urticaria has been described in patients with T1D and other AIDs, such as Hashimoto thyroiditis, hyperthyroidism, and Raynaud's phenomenon [159]. Among the reports in Literature, two children aged 12 and were diagnosed with thyroid disease, T1D, and chronic urticaria [160, 161]; one of the patients was started on omalizumab and successfully treated without changes in his glycemic control [160].

Although, the most common cutaneous manifestations are related to the metabolic consequences of T1D, patients with diabetes are also at higher risk of developing autoimmune skin conditions. It is important to consider the psychological impact of these cutaneous since, even in mild cases, they may represent an additional burden for the patients, especially for adolescents [37].

#### 2.3.6. Endocrine Disorders and Autoimmune Polyendocrine Syndromes (APSs)

Although, the coexistence of T1D and thyroid disease is frequently reported, T1D may also occur with other endocrine autoimmune conditions, usually in the set of APSs. APSs are a cluster of inherited clinical entities that involve the impairment of multiple endocrine glands due to loss of immune tolerance [162]. The clinical manifestations involve many of the conditions that we have previously discussed, but this time the dysimmune activation is explained by genetic mutations that involve the AIRE gene, leading a to lack of deletion of autoreactive T cells and to formation of dysfunctional T regs [162]. It is still unknown why some tissues are particularly susceptible in becoming the target of the immune response.

APS-2 occurs more frequently than APS-1, with an incidence of 1–2 : 1,000,000 per year [163]. T1D is one of the most common manifestations of APS-2, whereas it does not occur in APS-1, in which patients present AD, hypoparathyroidism, and chronic mucocutaneous candidiasis [164]. APS-2 is defined by the presence of AD (always present), in combination with autoimmune thyroid disease (70% of cases) and/or T1D (40–50) [164]. Patients may also develop autoimmune gastritis, alopecia, vitiligo, CD, and primary ovarian insufficiency [164]. The clinical presentation and diagnosis of these conditions in the context of APS-2 are the same as that of individual diseases. However, the overlap between AD and T1D may lead to hypoglycemic episodes because of impaired gluconeogenesis and greater insulin sensitivity, leading to a harder management of both diseases. Patients suffering from AD and T1D and who receive glucocorticoid replacement therapy have a lower need for basal insulin and an increased need for postprandial insulin compared to patients with T1D [31, 165]. In a patient with APS2, thyroid dysfunction may also alter the glucose tolerance, with the onset of hypoglycemia in patients with hypothyroidism and hyperglycemia in the ones with hyperthyroidism, respectively for a reduced and increased insulin need [164].

The coexistence of AD and T1D in the absence of APS-2 is rare, with a prevalence of T1D of 10%–14% in patients with AD [166], whereas 0.2%–0.4% of diabetic patients have AD [167]. The main autoantibodies involved in AD are anti-21-hydroxylase antibodies, which are present either in the isolate AD or in the set of APS-2 [31]. Patients with both conditions are at risk for increased incidence of the typical acute metabolic disorders relative to each condition, such as adrenal crisis and hypoglycemia [168]. These complications may be related to misalignment between the two treatments or a delay in diagnosis. Patients with the onset of AD may be asymptomatic or present mild symptoms (e.g., fatigue, nausea, appetite loss) initially, and slowly progress to an Addisonian crisis. Since, no screening procedures for AD are routinely recommended for patients with T1D, physicians need to be aware of the risk of developing such a condition, especially if the patient's history is positive for thyroid disease or severe infections [166]. An unexplained change in the glycemic trend, such as persistent hypoglycemic levels, may also be a reason to suspect the onset of AD [169].

Similarly to APSs, another genetic condition that leads to T1D and other AIDs is X-linked immune dysregulation polyendocrinopathy and enteropathy (IPEX), a disease characterized by a defect in the FOXP3 gene involved in Treg maturation [170]. IPEX is a rare syndrome (incidence of 1 : 1,000,000 per year) that leads to early onset T1D, autoimmune enteropathy, and eczema. Autoimmune thyroid disease, alopecia, hemolytic anemia, and thrombocytopenia can also occur. The first symptoms—usually related to the enteropathy (e.g., malabsorption syndrome, weight loss, failure to thrive)—manifest earlier than in APSs, and the condition can be fatal if not treated with immunosuppressive agents or bone marrow transplantation.

## 3. Conclusions

The rise in the incidence of AIDs among children warrants more awareness from pediatricians, since earlier diagnosis may significantly reduce the morbidity and improve the outcome for these patients. Every child with a diagnosis of AID should be monitored periodically, keeping into account the clinical performance achieved during therapy. The physician needs to be careful when facing signs or symptoms that may suggest the onset of another disorder, especially when symptoms such as weight loss and anemia may be easily overlooked or assumed to be caused by diseases that are already known.

Therefore, a better knowledge of the epidemiology of overlap between AIDs may guide and define the need for an early and/or periodic screening for such conditions. As it is good practice to check thyroid function as part of the diagnostic evaluation and monitoring for many AIDs (e.g., T1D, CD, and MS), or to screen for CD right after the diagnosis of T1D and again within 2 and 5 years if prior screening studies are negative, it is important to screen other conditions as soon as they appear clinically suggestive.

However, since most of the data about the incidence of comorbidities of T1D come from studies with small cohorts of patients, further and larger studies coming from different countries are needed to better assess the incidence of other disorders, before deciding if any of these may be worth screening periodically in children with diabetes.

Advances in the understanding of genetics and metabolomics may help us define the population to screen to precociously identify patients at risk and, hopefully, treat them in the preclinical period before it is too late. As supported by the clinical outcome of patients with comorbidities who are receiving disease-modifying drugs, the goal for the future of pediatric diabetes may be the early administration of immunosuppressive therapies, which can stop the dysimmune process before it destroys beta cells, with the same logic that is already guiding the treatment for other AIDs as JIA and MS [171]. These therapies also represent a useful tool to optimize disease control, with a double positive effect on T1D: on one hand, reducing disease flares, whose inflammatory state causes hyperglycemia and glucose variability; on the other, limiting the prescription of corticosteroids, which are the staple of the treatment of the acute phase of some of these AIDs (IBDs, JIA, SLE, MS, nephrotic syndrome…), but have a deleterious effect on glucose metabolism, leading to hyperglycemia and reduction of insulin sensitivity.

Finally, children affected by one or more AIDs need well-coordinated multidisciplinary care, and support is necessary for patients and their families.

Many steps forward are needed to assess the prevalence of these disorders, the genes involved, the optimal screening and to tailor treatment for each patient. However, a deeper understanding of the commonalities and differences between autoimmune conditions will accelerate progress, through the cross-fertilization of knowledge, toward an earlier diagnosis and more effective tailored management.

## Figures and Tables

**Table 1:** Prevalence of some of the most frequent autoimmune disorders in children with type 1 diabetes compared to the pediatric population.

	Prevalence in children with T1D	Prevalence in the pediatric population
Autoimmune gastritis	5%–10% [9, 10]	0.15% [11]
Celiac disease	4.6%–16% [12–16]	1.4% [17, 18]
Graves' disease	0.4%–4% [19, 20]	0.01% [21–23]
Hashimoto thyroiditis	4%–24% [19, 24–27]	1%–3% [28]
Juvenile idiopathic arthritis	0.19% [29, 30]	0.05% [29, 30]
Psoriasis	2%–8% [31–33]	0.5–1.7% [34, 35]
Vitiligo	1.6%–10% [36, 37]	0.09%–1% [38]

Of note, the estimated prevalence reflects the characteristics of the population studied.

**Table 2:** Potential environmental risk factors linked to autoimmune diseases in children.

UV light exposure	SLE, MS, T1D
Vitamin D deficiency	SLA, MS, CD, T1D
Hormones	MS, SLE
Obesity	T1D, JIA, MS, IBDs
Infections	*Streptococcus pyogenes*—Rheumatic fever *Cytomegalovirus*—SLE, T1D *Epstein-Barr Virus*—SLE, MS *Human Herpesvirus 6*—MS *Enterovirus, Rotavirus*—T1D *Helicobacter pylori*—Autoimmune gastritis *SARS—COVID-19*—T1D, CD, SLE, IBD, Transverse myelitis

Abbreviations: CD, celiac disease; IBD, inflammatory bowel diseases; JIA, juvenile idiopathic arthritis; MS, multiple sclerosis; SLE, systemic lupus erythematosus; T1D, Type−1 diabetes.
